# Biodiversity and distribution patterns of blooming jellyfish in the Bohai Sea revealed by eDNA metabarcoding

**DOI:** 10.1186/s12862-024-02224-3

**Published:** 2024-03-18

**Authors:** Lijing Ye, Saijun Peng, Yuanqing Ma, Wenjing Zhang, Lei Wang, Xiyan Sun, Chen Zhang, Munjira Yeasmin, Jianmin Zhao, Zhijun Dong

**Affiliations:** 1 Yantai University, 264005 Yantai, Shandong China; 2 Muping Coastal Environment Research Station, Yantai Institute of Coastal Zone Research, Chinese Academy of Sciences, 264003 Yantai, Shandong China; 3 University of Chinese Academy of Sciences, 100049 Beijing, China; 4 Shandong Key Laboratory of Marine Ecological Restoration, Shandong Marine Resource and Environment Research Institute, 264006 Yantai, Shandong China

**Keywords:** Environmental DNA metabarcoding, Jellyfish bloom, Biodiversity, Spatial distribution

## Abstract

**Background:**

The mass occurrence of scyphozoan jellyfish severely affects marine ecosystems and coastal economies, and the study of blooming jellyfish population dynamics has emerged in response. However, traditional ecological survey methods required for such research have difficulties in detecting cryptic life stages and surveying population dynamics owing to high spatiotemporal variations in their occurrence. The environmental DNA (eDNA) technique is an effective tool for overcoming these limitations.

**Results:**

In this study, we investigated the biodiversity and spatial distribution characteristics of blooming jellyfish in the Bohai Sea of China using an eDNA metabarcoding approach, which covered the surface, middle, and bottom seawater layers, and sediments. Six jellyfish taxa were identified, of which *Aurelia coerulea*, *Nemopilema nomurai*, and *Cyanea nozakii* were the most dominant. These three blooming jellyfish presented a marked vertical distribution pattern in the offshore regions. *A. coerulea* was mainly distributed in the surface layer, whereas *C. nozakii* and *N. nomurai* showed a upper-middle and middle-bottom aggregation, respectively. Horizontally, *A. coerulea* and *C. nozakii* were more abundant in the inshore regions, whereas *N. nomurai* was mainly distributed offshore. Spearman’s correlation analysis revealed a strong correlation between the eDNA of the three dominant blooming jellyfish species and temperature, salinity, and nutrients.

**Conclusions:**

Our study confirms the applicability of the eDNA approach to both biodiverstiy evaluation of blooming jellyfish and investigating their spatial distribution, and it can be used as a supplementary tool to traditional survey methods.

**Supplementary Information:**

The online version contains supplementary material available at 10.1186/s12862-024-02224-3.

## Background

Jellyfish, gelatinous cnidarians, are critical elements of marine ecosystems, and they greatly influence food webs and elemental fluxes [1]. Historically, jellyfish blooms have been considered occasional and episodic; however, in recent years, reports of such events have increased dramatically [2–4]. Mounting evidence indicates that these blooms cause losses in aquaculture and tourism, block nuclear power plants, and reduce fish catches [5, 6]. Therefore, increasing concerns about the population dynamics of gelatinous jellyfish have been raised [7–10]. However, jellyfish samples can be difficult to identify using traditional visual or plankton trawling methods, due to the fragility of their tissues, their metagenic life cycles, and the existence of cryptic species. In addition, the uneven vertical distribution of jellyfish in the water column poses a challenge to traditional field-based ecological surveys [11–13]. A fast, highly recognizable, and accurate technique is urgently needed as a supplement (or even alternative) to traditional approaches.

In this respect, environmental DNA (eDNA) metabarcoding, an emerging method, combines traditional ecological approaches with in-depth molecular methods and advanced computational tools to investigate biodiversity and biomass [14]. The term, eDNA is defined as the DNA fragments present in environmental samples, including whole cells, extracellular DNA, and potentially whole organisms [15, 16]. This technique has been effectively used in biodiversity surveys, endangered species tracking, invasive species detection, ancient ecosystem reconstruction, pollution prediction, and diet analyses [16–22]. However, its application in the analysis of targeted jellyfish taxa remains relatively limited. Nevertheless, several published studies have assessed and demonstrated the effectiveness of eDNA metabarcoding in jellyfish (Medusozoa) detection [23–25]. DNA metabarcoding analysis of fecal and intestinal samples from multiple species has been used to indirectly detect the presence of jellyfish, possibly reflecting their population dynamics [26, 27]. Furthermore, eDNA techniques have enormous potential for monitoring the vertical distribution patterns of jellyfish [13, 28]. Thus, using this powerful eDNA tool to conduct jellyfish taxa assessment in hotspots may provide a reliable foundation for the monitoring and prevention of jellyfish blooms.

The Bohai Sea, a semi-enclosed and marginal sea in China, is characterized by abundant fishery resources, intense human activity, and increasing ocean engineering. Recently, jellyfish assemblages and blooms have been frequently reported there, especially in the harbors and coastal waters [29–31]. These blooms block coastal nuclear power plants, cause an imbalance in marine ecosystems, and are associated with a decline in economic fishery production [2, 32]. This study aimed to develop an effectively indicative tool for investigating jellyfish, which will contribute to constructing monitoring systems of jellyfish blooms. In this respect, we investigated the diversity and distribution of blooming jellyfish during a periodic jellyfish outbreak in August 2022 in the Bohai Sea using eDNA metabarcoding based on the 16S rRNA gene, and then conducted a correlation analysis between the distribution of dominant jellyfish and environmental factors.

## Methods

### Sample collection

**Fig. 1:**
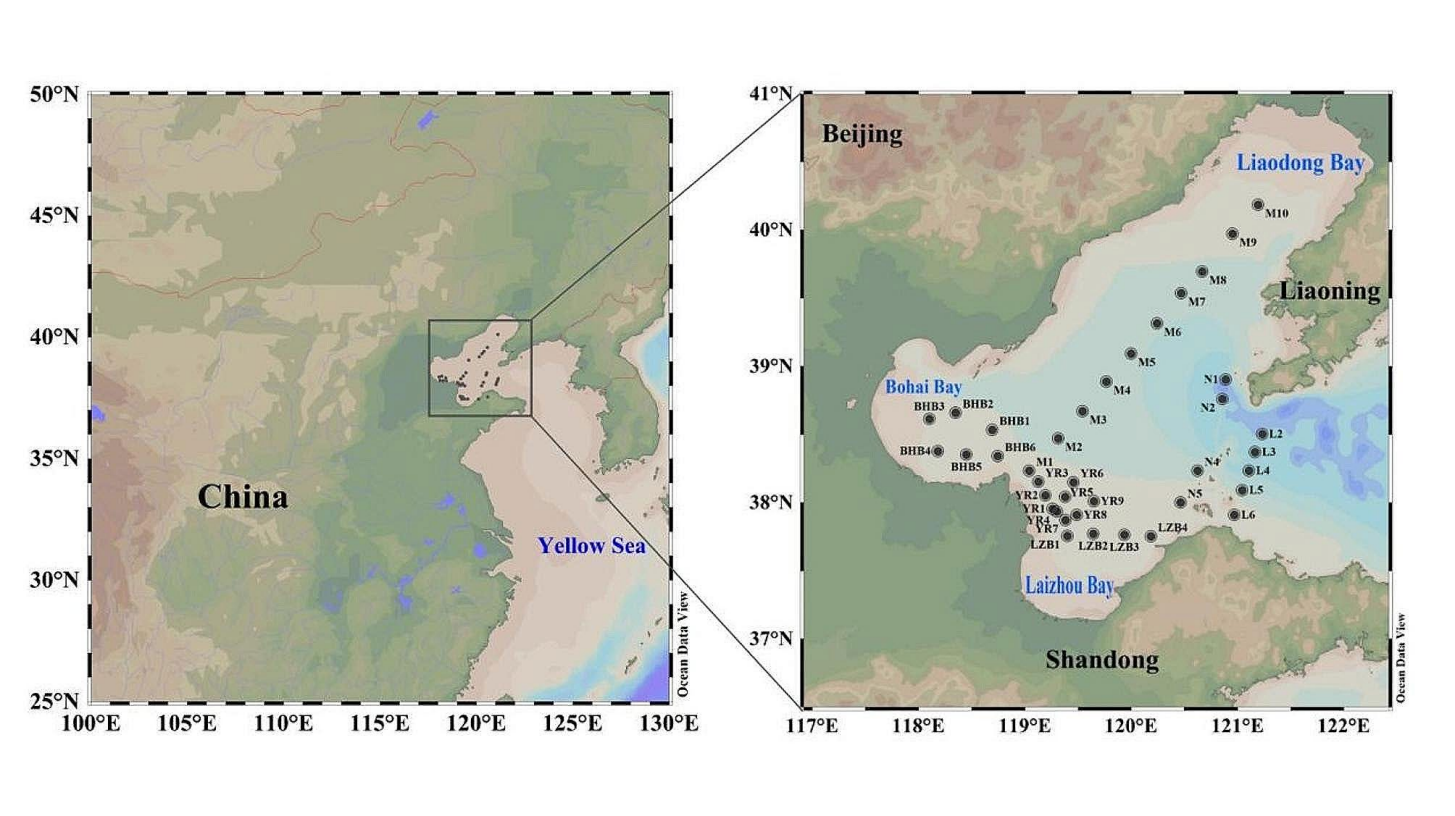
Maps of sampling stations in the Bohai Sea. *Note* The maps in the figure were obtained from Ocean Data View software (Reiner Schlitzer, Alfred Wegener Institute, Bremerhaven, Germany)

The investigation was conducted at 38 stations in the Bohai Sea in August 2022 (118°06.222′-121°13.915′ E, 37°45.278′-40°11.093′ N); six stations in the Bohai Bay (BHB1-6), four stations in the Laizhou Bay (LZB1-4), ten stations in the central Bohai Sea area (M1-10), nine stations at the Yellow River estuary (YR1-9), four stations near the Miaodao Archipelago (N1-2, N4-5), and five stations in the Bohai Strait (L2-6) (Fig. 1). We categorized those located in the Bohai Bay, the Laizhou Bay, the Yellow River estuary, and M1-3 in the central Bohai Sea as inshore stations; those in the Miaodao Archipelago, the Bohai Strait, and M4-10 in the central Bohai Sea were categorized as offshore stations. Seawater samples were collected from three depths (surface, middle, and bottom layers; the average sampling depths are shown in Table 1); sediment samples were collected separately. A total of 114 seawater and 24 sediment samples were obtained.

**Table 1:** Environmental characteristics of the Bohai Sea (mean ± SE)

Layer	PO_4_^3−^ (mg/L)	NH_4_^+^ (mg/L)	NO_2_^−^ (mg/L)	SiO_3_^2−^ (mg/L)	NO_3_^−^ (mg/L)	Chl a (µg/L)	Temp (T) (℃)	Salinity (S) (ppt)	Depth (D) (m)
Surface	0.0038 ± 0.0017^**b**^	0.0207 ± 0.0161^**b**^	0.0296 ± 0.0286	0.2927 ± 0.2163	0.1188 ± 0.1336	3.95 ± 2.61	26.15 ± 1.41^**a**^	28.27 ± 1.32	2.98 ± 0.38^**c**^
Middle	0.0048 ± 0.0021^**a**^	0.0289 ± 0.0161^**a**^	0.0310 ± 0.0213	0.3131 ± 0.1704	0.1103 ± 0.0846	2.91 ± 2.73	24.35 ± 3.02^**b**^	28.76 ± 1.32	10.97 ± 4.85^**b**^
Bottom	0.0044 ± 0.0019^**ab**^	0.0276 ± 0.0137^**ab**^	0.0358 ± 0.0233	0.3103 ± 0.1765	0.1242 ± 0.1011	3.02 ± 2.53	24.17 ± 3.16^**b**^	28.84 ± 1.28	21.24 ± 13.89^**a**^

*Note* The superscript letters represent the statistical differences between environmental characteristics at different water depths

The seawater samples were collected from the surface, middle, and bottom seawater at each station using a water sampler integrated with a Sea-Bird conductivity-temperature-depth (CTD) profiler (Sea-Bird Scientific Inc., the United States). One liter of seawater was filtered through 0.7 μm GF/F (glass fiber filters) membranes (Whatman, Maidstone, UK), which were then stored in 2-mL sterile cryopreservation tubes (Beyotime, Shanghai, China). Sediment samples were collected using a bottom sampler and stored in 50-mL sterile centrifuge tubes with a sterile disposable syringe. All membrane and sediment samples were temporarily frozen in liquid nitrogen and quickly transferred to a -80 ℃ refrigerator when returned to the laboratory. To avoid cross-contamination between samples, all devices used for sample collection and filtration were bleached with 10% sodium hypochlorite and washed at least twice with Milli-Q water before sampling. In addition, a negative blank control was collected to examine for potential contamination, 1 L of distilled water was filtered at each station and the membrane was preserved.

The temperature (T), salinity (S), and depth (D) at different depths at each station were recorded using the CTD profiler. The seawater used to measure nutrients and chlorophyll *a* (Chl *a*) was obtained during the collection of the above seawater samples. Subsequently, 500 mL seawater was filtered through a 0.7 μm GF/F membrane (Whatman, Maidstone, UK) [12, 33] for the estimation of Chl *a*. Chl *a* was extracted with 90% acetone at -20 ℃ in the dark for 12 h, after which it was measured using a fluorophotometer (Trilogy, Turner Designs, USA) [34]. To measure the nutrient concentrations, 50 mL of seawater was filtered through a 0.45 μm polyethersulphone membrane. Phosphate (PO_4_^3−^), silicate (SiO_3_^2−^), nitrate (NO_3_^−^), nitrite (NO_2_^−^), and ammonia (NH_4_^+^) levels were determined using a QuAAtro automatic analyzer (SEAL Inc., Germany) [35].

### eDNA extraction

eDNA on the GF/F filter membranes was extracted using a DNeasy Blood & Tissue Kit (Qiagen, Hilden, Germany) according to the protocol described in Takahashi et al. (2020) [33], with slight adjustments. A single membrane was placed in the suspended part of a Salivette tube (Sarstedt, Nümbrecht, Germany) and a 440 µL mixed solution containing 40 µL protease K and 400 µL AL lysis buffer was added to the membrane. After incubation for 1 h at 56 ℃, centrifugation was performed at 5000 × *g* for 3 min to collect the pyrolysis liquid. Subsequently, 200 µL TE buffer was added onto the membrane, followed by repeated centrifugation. A mixed solution consisting of 200 µL AL buffer and 600 µL anhydrous ethanol was then added to the collected liquid, and the mixture was transferred to a spin column, which was operated according to the manufacturer’s instructions. The eDNA was then eluted in 80 µL AE buffer and finally stored at -20 ℃. A blank filter membrane as a negative control was also subjected to all the steps mentioned above to detect possible contamination during extraction.

eDNA was extracted from the sediments using the DNeasy PowerSoil Pro Kit (Qiagen, Hilden, Germany). Approximately 0.25 g of sediment per sample was weighed for extraction, and the extraction steps followed the manufacturer’s instructions. The eDNA was then eluted with 80 µL of Solution C6 and stored at -20 ℃. A 0.25 g Milli-Q water sample was included as the negative control to detect contamination during this process.

### eDNA metabarcoding and sequence processing

The 16S rRNA gene fragments of extracted eDNA (*n* = 138) were amplified using cnidarian-universal primers: 16 S-L 5´-GACTGTTTACCAAAAACATA-3´ and 16 S-H 5´-CATAATTCAACATCGAGG-3´ [36]. PCR amplification was performed on an ABI GeneAmp® 9700 PCR instrument (Applied Biosystem, Waltham, Massachusetts), using a 20 µL reaction system of TransStart Fastpfu DNA Polymerase (TransGen AP221-02) that included 4 µL 5 × FastPfu Buffer, 2 µL 2.5 mM dNTPs, 0.8 µL each of forward and reverse primers with barcodes (5 µM), 0.4 µL FastPfu Polymerase, 0.2 µL BSA, 2 µL template DNA, and 9.8 µL double distilled H_2_O. The thermal conditions for the PCR reaction were as follows: initial denaturation at 95 ℃ for 3 min and 37 cycles of 95 ℃ for 30 s, 60 ℃ for 30 s, and 72 ℃ for 45 s, followed by a final extension executed at 72 ℃ for 10 min. Each eDNA sample was amplified three times, and three replicates from the same sample were mixed and analyzed using 2% (w/v) agarose gel electrophoresis. The PCR products recovered using the AxyPrepDNA gel recovery kit were quantified using a QuantiFluor^TM^-ST Blue Fluorescence Quantification System (Promega, Beijing, China) and normalized to equimolar amounts. Amplicon libraries were constructed using TruSeq™ DNA Sample Prep Kit (Illumina). Paired-end sequencing (2 × 300 bp) was performed using an Illumina MiSeq platform (Majorbio Bio-Pharm Technology Co., Ltd., Shanghai, China).

Paired-end reads were merged with FLASH v 1.2.11 [37] to obtain spliced sequences. The spliced sequences were subjected to quality control and filtering to obtain high-quality clean reads using QIIME v 1.9.1 [38, 39]. Chimeric sequences were detected and removed using the UCHIME algorithm [40]. Sequences with ≥ 97% similarity were assigned to the same operational taxonomic units (OTUs), and the representative sequence for each OTU was screened for further annotation using the UPARSE software [41]. Taxonomic annotation was performed using the Nucleotide Sequence Database (nt_v20210917) from the NCBI database based on BLAST (E-value = 10^− 5^). Only OTUs classified as Metazoa were retained, and the sequence numbers were normalized using the standard of the sample with the fewest sequences.

### Data processing and statistical analysis

Maps of the sampling stations were visualized using Ocean Data View (Reiner Schlitzer, Alfred Wegener Institute, Bremerhaven, Germany). One-way analysis of variance (ANOVA) was used to identify the significance of environmental factors and relative read abundance of jellyfish at different stations and water depths. The Student’s *t* test was performed to analyze the significance of the relative read abundance of jellyfish in the seawater and sediment samples. The neighbor-joining phylogenetic tree of the six jellyfish taxa detected in this study was constructed using MEGA v11.0.11. Spearman’s correlation analysis between the relative read abundance of jellyfish taxa and environmental factors in the Bohai Sea was performed using the Omicshare platform (https://www.omicshare.com/tools/).

## Results

### Environmental characteristics

**Fig. 2:**
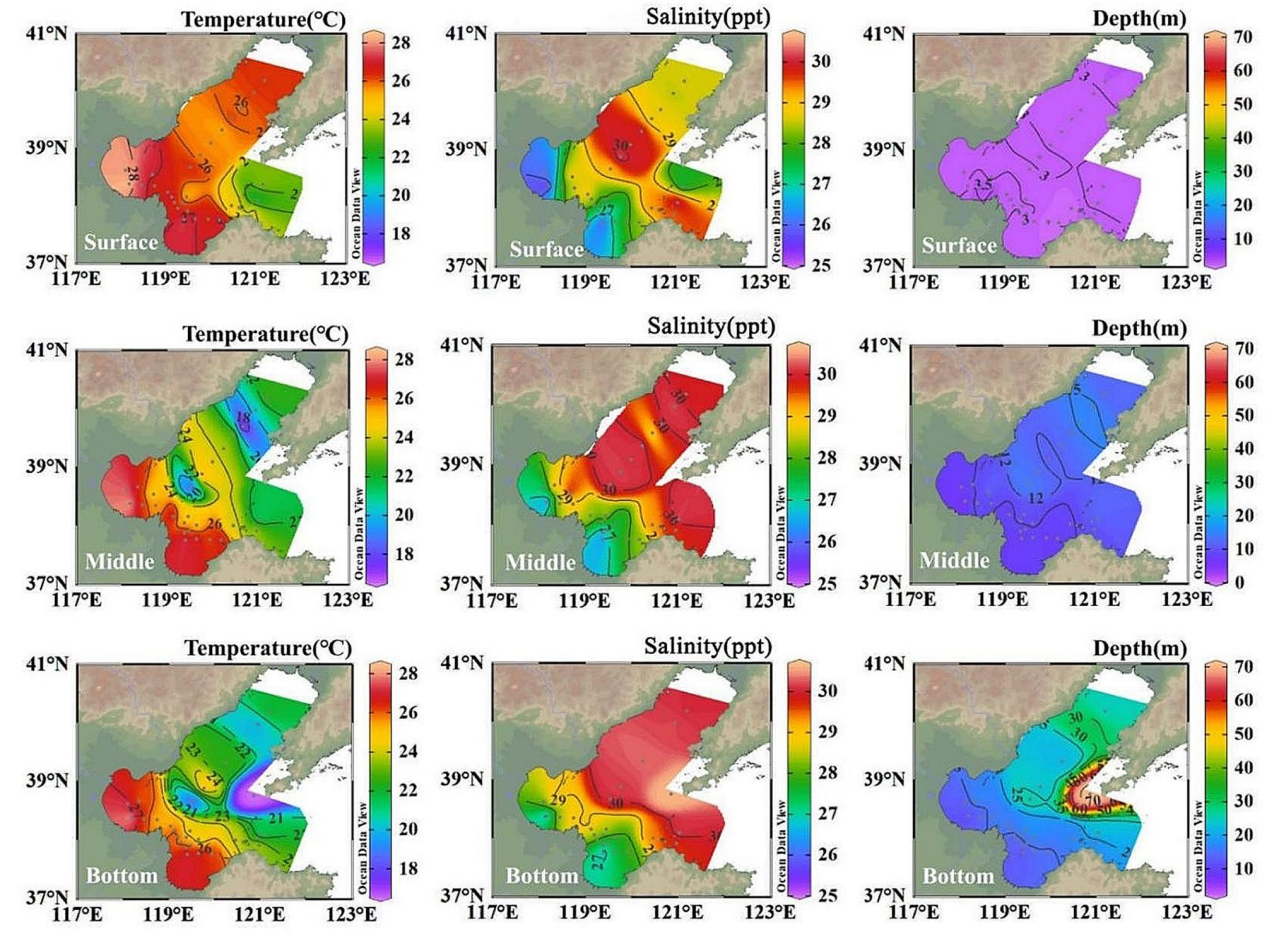
Variations in temperature, salinity, and depth in the Bohai Sea in August 2022. *Note* The maps in the figure were obtained from Ocean Data View software (Reiner Schlitzer, Alfred Wegener Institute, Bremerhaven, Germany)

**Fig. 3:**
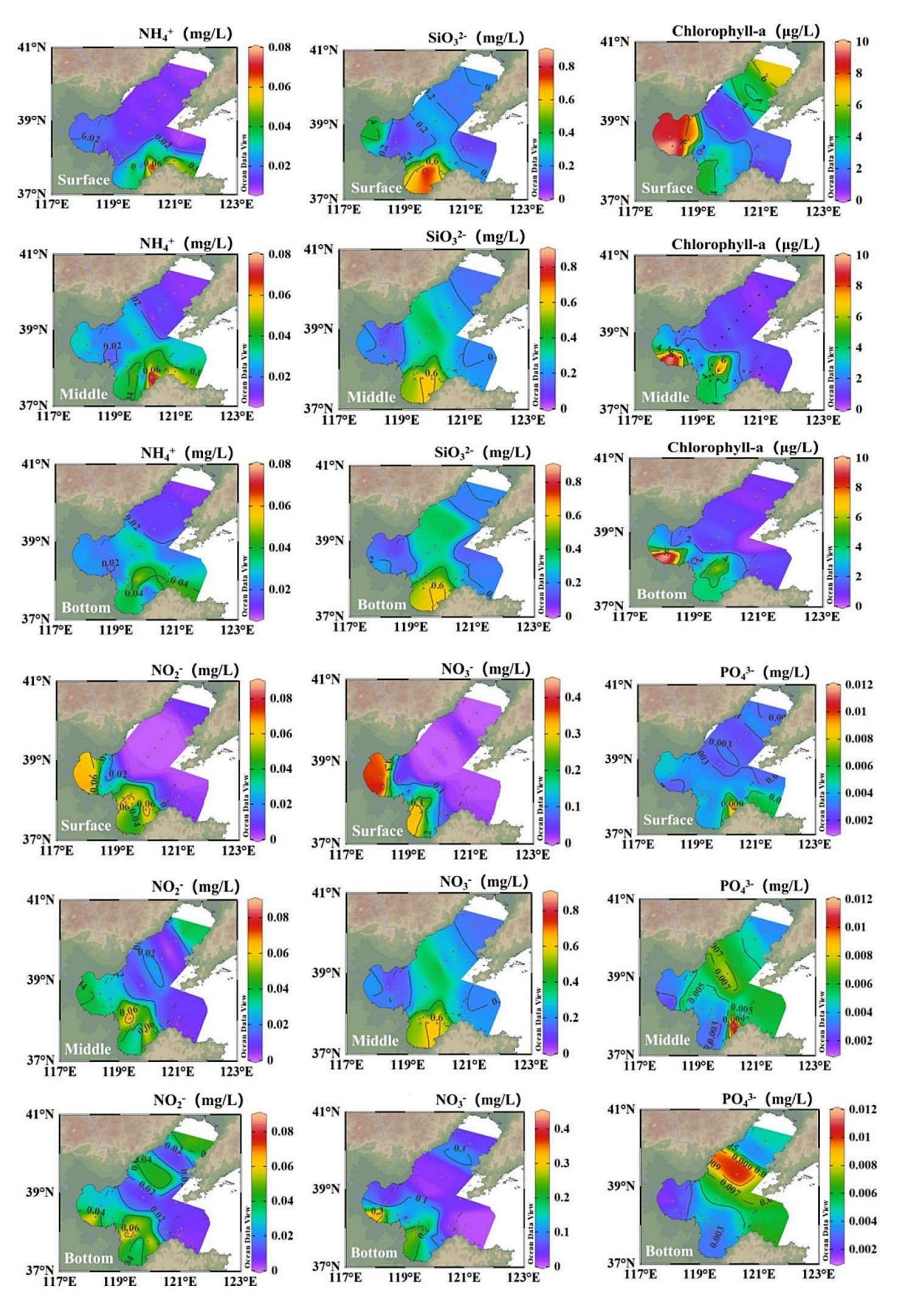
Variations in nutrient and chlorophyll-*a* concentrations in the Bohai Sea in August 2022. *Note* The maps in the figure were obtained from Ocean Data View software (Reiner Schlitzer, Alfred Wegener Institute, Bremerhaven, Germany)

The temperature, salinity, Chl *a*, and nutrient concentration distributions at different depths at the 38 stations are shown in Table 1; Figs. 2 and 3. The sampling depth ranged from 2.45 to 70.90 m. The surface temperatures were significantly higher than those of the middle (ANOVA, *P* < 0.05) and bottom (ANOVA, *P* < 0.01) depths. The inshore seawater exhibited higher temperatures and lower salinity than those of the offshore seawater in the middle and bottom layers (ANOVA, *P* < 0.01), whereas those at sea surface were similar. Additionally, the inshore concentrations of NO_3_^−^ and NO_2_^−^ decreased with seawater depth (ANOVA, *P* < 0.05), whereas PO_4_^3−^ maintained a low concentration distribution at all three seawater depths. The concentration of PO_4_^3−^ increased from the surface to the bottom in the central Bohai Sea area (ANOVA, *P* < 0.05). Furthermore, the inshore concentrations of NH_4_^+^, SiO_3_^2−^, and Chl *a* were significantly higher than those in the offshore seawater (ANOVA, *P* < 0.05).

### eDNA metabarcoding for jellyfish biodiversity detection

Ninety-eight amplicon libraries, derived from 28 surface seawater, 28 middle seawater, 27 bottom seawater, and 15 sediment samples across 33 stations, were successfully constructed from the 138 eDNA samples using cnidarian universal primer pairs. The 16S rRNA gene amplicon sequencing generated 5,120,633 high-quality filtered sequences. In total, 29 OTUs were clustered in Metazoa with 97% sequence similarity. Sixteen of these were classified as the phylum Cnidaria, covering three classes, 13 orders, 13 families, and 13 genera. Six medusozoan taxa were identified, including the scyphozoans *Aurelia coerulea*, *Nemopilema nomurai* and *Cyanea nozakii*, and the hydrozoans *Eirene ceylonensis*, *Ectopleura crocea*, and *Craspedacusta sowerbii* (Table 2). The identification rate of medusozoan taxa was above 99.42%, confirming the reliability of the taxonomic annotation (Table 2). Subsequently, a neighbor-joining phylogenetic tree of the six medusozoan species was constructed (see Additional file 1), and the genetic relationships between the six species were revealed.

**Table 2:** Summary of jellyfish taxa identified by eDNA metabarcoding based on 16S rRNA gene sequences

Phylum	Class	Family	Species	Best match in NCBI	
				Identity	Accession
Cnidaria	Scyphozoa	Rhizostomatidae	*Nemopilema nomurai*	100.00%	JX845343.1
		Ulmaridae	*Aurelia coerulea*	100.00%	MZ061800.1
				99.42%	OP458507.1
		Cyaneidae	*Cyanea nozakii*	100.00%	MW832752.1
	Hydrozoa	Olindiidae	*Craspedacusta sowerbii*	100.00%	MK600507.1
		Eirenidae	*Eirene ceylonensis*	99.63%	HM053550.1
		Tubulariidae	*Ectopleura crocea*	100.00%	MG811598.1

### Spatial distribution pattern of the blooming jellyfish

**Fig. 4:**
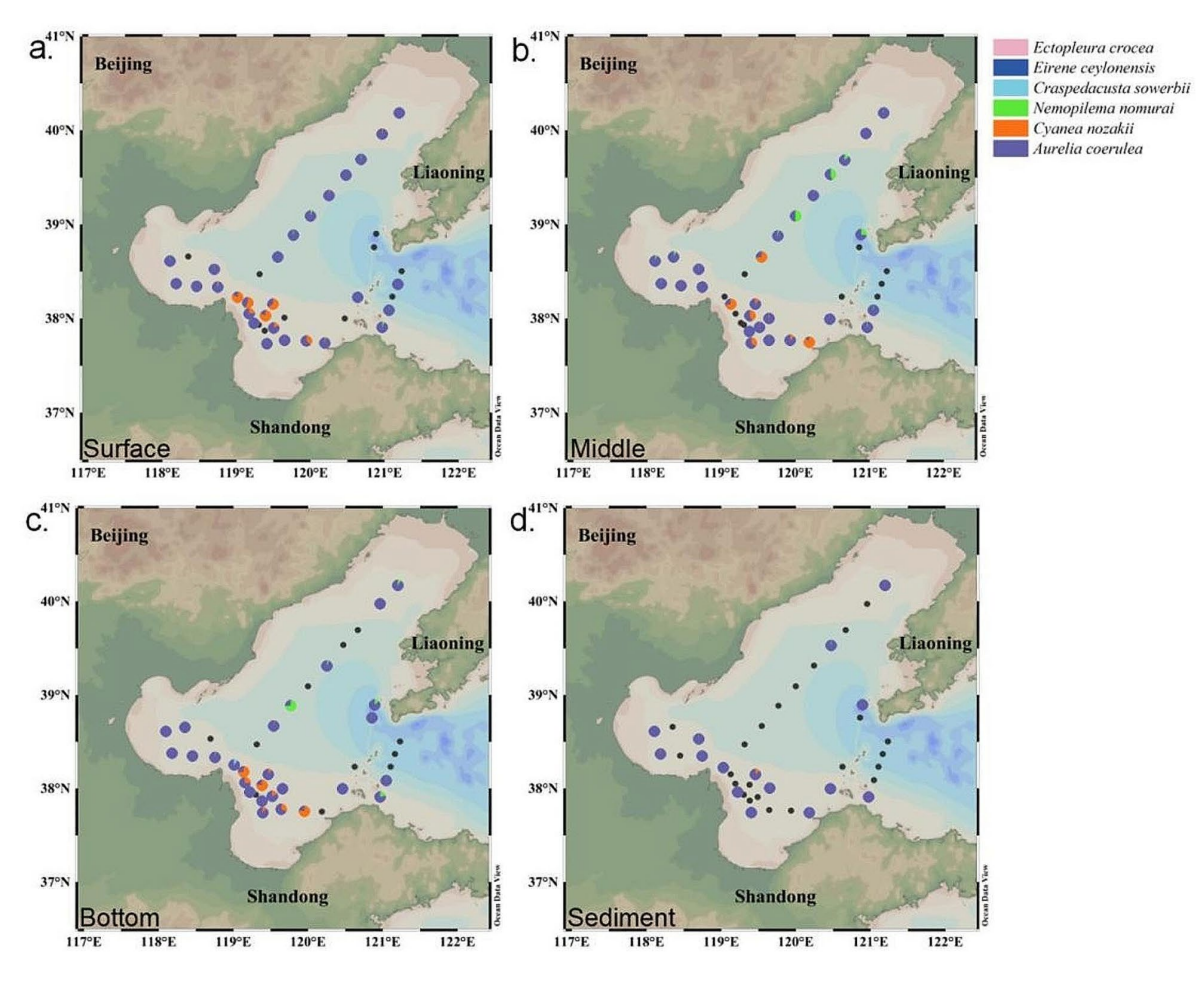
Spatial variations in the relative read abundance of six jellyfish taxa in the Bohai Sea in August 2022. *Note ***a**–**d**: Taxonomic composition and relative read abundance of jellyfish in eDNA from surface seawater (**a**), middle seawater (**b**), bottom seawater (**c**) and sediments (**d**). Jellyfish species represented by the distinct colors in the pie plots are described in the legend on the right. The black dots indicate that no jellyfish was detected in eDNA from the station. The maps in the figure were obtained from Ocean Data View software (Reiner Schlitzer, Alfred Wegener Institute, Bremerhaven, Germany)

*A*. *coerulea* was detected in 98 eDNA samples from the Bohai Sea during the survey period and was the most abundant jellyfish species in 86 eDNA samples, with relative read abundance 54.48% to 100% (Fig. 4). Among 22 eDNA samples from 12 stations, *A. coerulea* was the only jellyfish species identified (Fig. 4). Ten of the eDNA samples were dominated by *C. nozakii*, and all relative read abundance were 57.52% or greater; the highest relative read abundance was detected in the middle seawater of LZB4 (88.20%) (Fig. 4). *N*. *nomurai* was detected in 42 eDNA samples and was the most abundant in the middle seawater of M5 (55.20%) and bottom seawater of M4 (74.03%) (Fig. 4). Interestingly, eDNA samples with high *C. nozakii* or *N. nomurai* abundance (relative read abundance > 10%) were not coincident, and there was a low overlap in the spatial distribution of these two jellyfish species (Fig. 4). Moreover, *E. ceylonensis*, *E. crocea*, and *C. sowerbii*, which are rare species, were only detected in three, one, and two eDNA samples, respectively, and with a low relative read abundance (relative read abundance ≦ 10%) (Fig. 4). Therefore, the spatial distribution pattern analysis of jellyfish mainly focused on the three dominant jellyfish taxa (*A. coerulea*, *C. nozakii*, and *N. nomurai*).

**Fig. 5:**
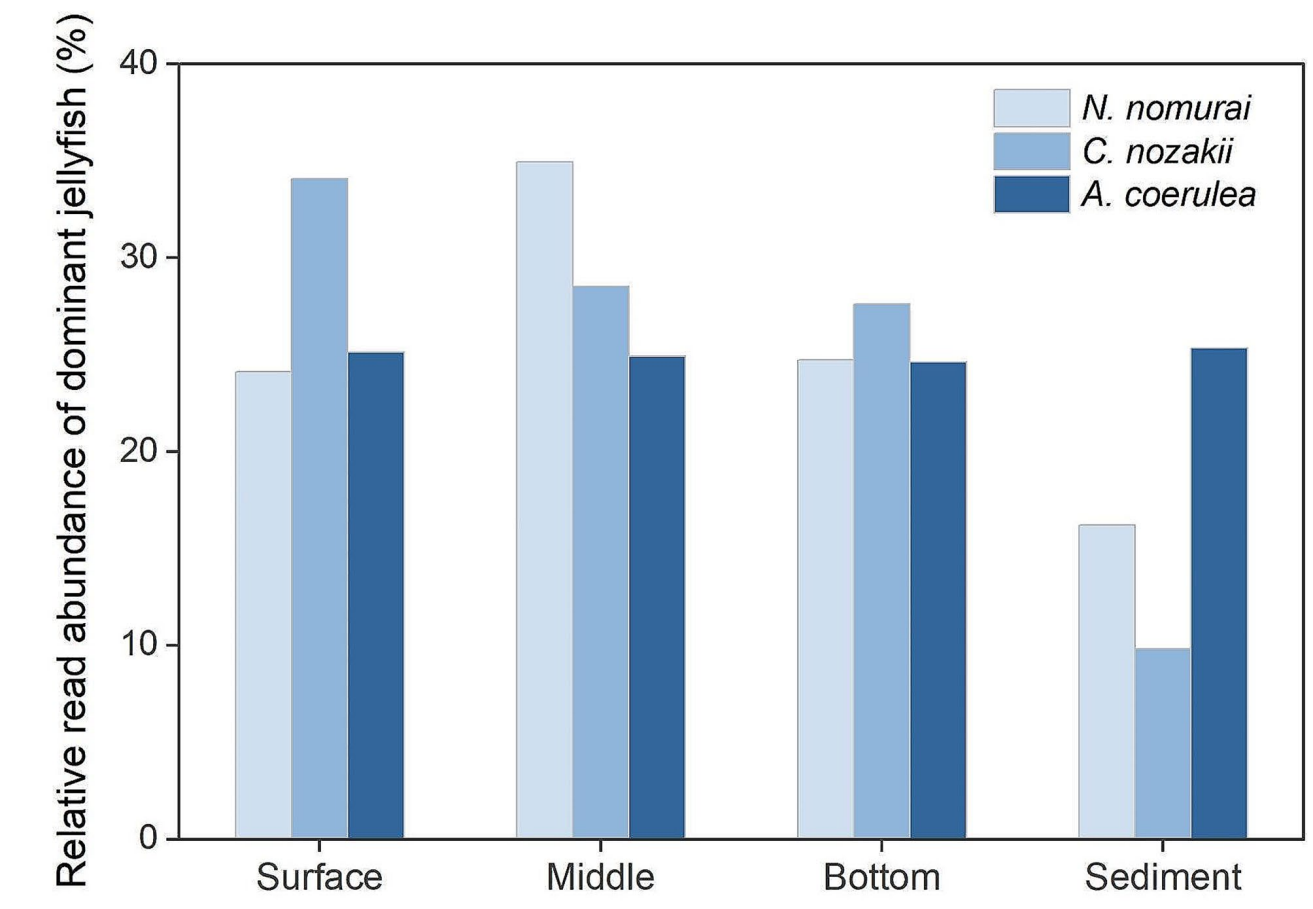
Vertical variations in the relative read abundance of dominant jellyfish taxa

Overall, in the surveyed Bohai Sea, the relative distribution proportions of *A. coerulea*, *C. nozakii*, and *N. nomurai* at different seawater depths were generally uniform. The eDNA of *C. nozakii* was more abundant in the surface layer, whereas that of *N. nomurai* was relatively aggregated in the middle layers (Fig. 5). *A*. *coerulea* displayed no significant difference in relative read abundances between sediments and seawater, while the relative read abundances of *C. nozakii* and *N. nomurai* were higher in seawater than in sediments (Student’s *t* test, *P* < 0.01; Fig. 5). Furthermore, marked differences between three jellyfish taxa in a vertical direction were revealed in seawater samples of partial regions. The relative read abundance of *A. coerulea* in the surface layer was significantly higher than that in the middle and bottom layers of the offshore stations (ANOVA, *P* < 0.05; Fig. 4a-c). The relative read abundance of *C. nozakii* in the offshore regions peaked in the middle layer and was significantly higher than that in the surface layer (ANOVA, *P* < 0.05; Fig. 4a-c). The eDNA of *N. nomurai* presented a middle-bottom aggregation at several offshore stations with a relative read abundance > 10% (M4, M5, M7, M8, N1, and L6), and was significantly more abundant in the bottom layer than in the surface layer (ANOVA, *P* < 0.05; Fig. 4a-c). Conversely, the difference between the vertical abundance of *A. coerulea*, *C. nozakii*, and *N. nomurai* was not significant in the inshore seawater (ANOVA, *P* > 0.05; Fig. 4a-c). In sediments, *A*. *coerulea* was the absolute dominant species, with a mean relative read abundance of 98.74% (Fig. 4d).

In terms of the horizontal distribution, the eDNA of *A. coerulea* was dominant in BHB, with a significantly higher abundance than in LZB, M, and YR (ANOVA, *P* < 0.05; Fig. 4). The eDNA of *C. nozakii* was mainly gathered in LZB and YR, as opposed to in BHB and the offshore seawater (ANOVA, *P* < 0.01; Fig. 4). The eDNA of *N. nomurai* was prevalent in M, where its relative read abundance was significantly higher than that in the inshore regions, including BHB, LZB, and YR (ANOVA, *P* < 0.05; Fig. 4).

### Correlation analysis between blooming jellyfish and environmental factors

**Fig. 6:**
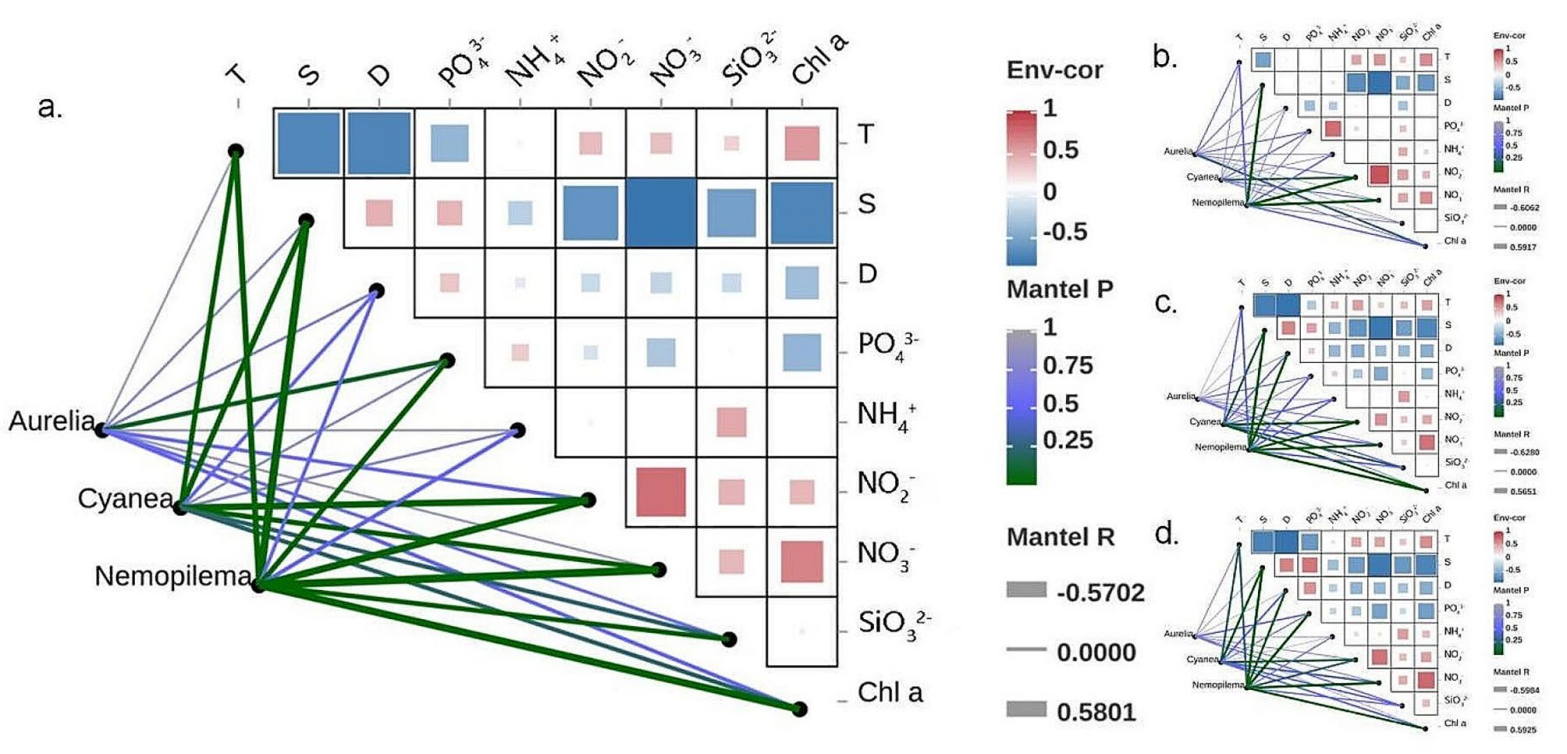
Spearman correlation analysis between the relative read abundance of dominant jellyfish taxa and environmental factors (**a**) in all 98 eDNA samples, and (**b**–**d**) in samples taken from the surface, middle, and bottom layers, respectively. *Note* A network heatmap of correlations between the relative read abundance of dominant jellyfish taxa and environmental factors. The color of the blocks in the right heatmap indicates a positive or negative correlation coefficient between different environmental factors, and the size of the blocks indicates the absolute value of correlation coefficients. The thickness of the lines indicates the strength of the correlations between the relative read abundances of the three dominant jellyfish species and environmental factors (Mental R), and the color of the lines indicates the degree of significance (Mental P)

Spearman correlation analysis showed that the relative read abundance of *A. coerulea* was significantly negatively correlated with the PO_4_^3−^ content (*P* < 0.05; Fig. 6a). The relative read abundance of *C. nozakii* was positively correlated with NO_3_^−^, NO_2_^−^, T, SiO_3_^2−^, and Chl *a* but negatively correlated with S (*P* < 0.05; Fig. 6a). Conversely, the relative read abundance of *N. nomurai* was negatively correlated with NO_3_^−^, NO_2_^−^, T, SiO_3_^2−^, and Chl *a* but positively correlated with PO_4_^3−^ and S (*P* < 0.01; Fig. 6a). The concentration of NH_4_^+^ was also negatively correlated with the relative read abundance of *N. nomurai* (*P* < 0.05; Fig. 6a).

Vertically, there were differences between the correlations of the three jellyfish species and environmental factors. A significantly positive correlation between the relative read abundance of *A. coerulea* and the Chl *a* concentration was detected in the surface seawater (*P* < 0.05; Fig. 6b). The relative read abundance of *C. nozakii* was positively correlated with NO_2_^−^ content at all three seawater depths, but negatively correlated with S in the middle and bottom layers (*P* < 0.05; Fig. 6b–d). In the middle and bottom layers, the relative read abundance of *N. nomurai* was positively correlated with S and PO_4_^3−^, but negatively correlated with NO_3_^−^, NO_2_^−^ and Chl *a* (*P* < 0.05; Fig. 6b–d).

## Discussion

As a new marine ecological survey tool, eDNA-based techniques are non-invasive, environmentally friendly, and accurate, and they are expected to play an essential role in optimizing ecological studies of jellyfish [42–44]. Recent studies have highlighted the great potential of eDNA-based techniques in biodiversity investigation, spatial distribution detection, and early life history stage monitoring of jellyfish [45–48]. Metabarcoding, as one of the major eDNA-based techniques, has obvious advantages in providing broad-scale distribution data for multiple species from a single analysis and detecting unknown biodiversity previously not recorded, including “unexpected” invasive or non-native species [21, 23, 28, 49]. Here, the eDNA metabarcoding was used as an indicator of the spatial distribution of jellyfish in the Bohai Sea. Six jellyfish species were identified in this study, of which *A. coerulea*, *C. nozakii* and *N. nomurai* were the dominant jellyfish taxa, which is consistent with the results of traditional ecological surveys [50–52].

### Indicator of the spatial distribution of the blooming jellyfish by eDNA

It is important to determine the vertical distribution of medusae to study their trophic interactions, vertical migration behavior, and spatiotemporal changes under global changes [11, 53, 54]. However, traditional sampling using plankton nets cannot effectively demonstrate the vertical distribution of jellyfish in shallow sea due to the trawling depths employed, the limited collection efficiency, and the damage caused to the fragile bodies of gelatinous organisms [55, 56]. In recent years, underwater imaging and acoustic equipment have been used to monitor the vertical distribution of jellyfish [53, 54, 57, 58]; however, these depend on diving and require large and expensive devices. In the current study, the distribution of three dominant jellyfish species, *A. coerulea*, *N. nomurai*, and *C. nozakii*, in the typical layers were indicated by eDNA metabarcoding, the results of which were relatively easier to realize.

We found that more eDNA from *A. coerulea* was detected in the surface layer of offshore seawater than in the middle and bottom layers. *N. nomurai* and *C. nozakii* were preferentially aggregated in the middle-bottom and upper-middle layers, respectively. These results are consistent with previous findings obtained using traditional methods. There are seasonal variations in the vertical distribution of *Aurelia* sp., and the study of Barz et al. (2005) found that most *A. aurita* were distributed in the upper layer in August [59]. Kim et al. (2016) found that approximately 93% of *N. nomurai* individuals were distributed within a water depth of 10–40 m [57]. It is of note that in this survey, there was a significant difference in temperature between the surface and the middle and bottom layers in a vertical direction, which indicates the existence of a possible thermal stratification in the Bohai Sea. The existence of thermal stratification may thus affect the vertical diffusion range of eDNA. Littlefair et al. (2021) demonstrated that eDNA signals show strong seasonal stratification during summer that closely reflects the thermal preference of fishes [60]. Therefore, the vertical distribution patterns of eDNA may also reflect the thermal preference of these three blooming jellyfish.

Horizontally, *A. coerulea* and *C. nozakii* were more abundant in the inshore regions than in the offshore regions, and this result is consistent with those of several previous trawl surveys on these two species [61, 62]. *N*. *nomurai* aggregations were mainly distributed in offshore areas, and similar results also were detected in an ecological investigation [50].

The relative read abundance of the three dominant jellyfish taxa in the sediments were significantly different from those in the seawater, indicating a difference between the eDNA preservation of the two media. Due to the low degradation of DNA in sediments, it is speculated that sediment eDNA reflects resident organisms and historical events rather than transient events [63–65]. Therefore, the eDNA analysis of sediments and seawater may contribute to different research purposes.

Although possible interference from eDNA released by the decaying jellyfish sinking to the bottom cannot be completely excluded in this study, the eDNA collected in this study is more likely to come from live jellyfish rather than dead individuals considering that our sampling time is not autumn and winter when jellyfish decay events occur frequently in the Bohai Sea [66].

### The response of blooming jellyfish to environmental factors in the Bohai Sea

The Spearman correlation analysis showed a significantly positive relationship between the relative read abundance of *C. nozakii* and both Chl *a* and every nutrient tested for. It also indicated a significantly positive relationship between the relative read abundance of *N. nomurai* and salinity, while negative correlations were determined between *N. nomurai* and NO_3_^−^, NO_2_^−^, temperature, SiO_3_^2−^, and Chl *a*, and between *C. nozakii* and salinity (Fig. 6). These results verified the preference of *C. nozakii* in the rich nutrient, highly productive, and low salinity inshore water, and the preference of *N. nomurai* in low salinity and low nutrient offshore environments. Notably, the relative read abundance of *N. nomurai* was positively correlated with PO_4_^3−^, which was likely the result of excretion of PO_4_^3−^ by *N. nomurai* themselves [67].

The relative read abundance of *A. coerulea* in the BHB was significantly higher than that in the LZB and other offshore regions, and showed less correlation with environmental factors in this study. Dong et al. (2012) also found that the spatial distribution of *A. coerulea* was less correlated with environmental variables in the Sishili Bay, and the intense construction of coastal aquaculture rafts may provide suitable habitats for their early growth [68]. In a survey of large jellyfish in the Bohai Sea in 2018, *A. coerulea* was found to peak in the BHB [69], particularly in an area that was used for marine utilization and human activities, including aquaculture and harbor engineering [70]. Therefore, the greater polyp habitat availability in the BHB could be associated with the high abundance of abundance of *A. coerulea* in this area, and water dynamics and currents may contribute to its patchy distribution in this region [71].

## Conclusions

The biodiversity and vertical and horizontal distribution patterns of blooming jellyfish in the Bohai Sea were investigated using eDNA metabarcoding in August 2022. The eDNA-based results corresponded to traditional findings of previous studies, confirming the feasibility of the eDNA approach for blooming jellyfish monitoring and population surveys over a wide range of scales. The current study highlights the advantages of eDNA metabarcoding as a tool for further revealing the vertical distribution of blooming jellyfish. However, limited by the diffusivity and instability of eDNA, the combined use of eDNA-based techniques and traditional ecological survey tools would contribute to making precise estimates of biodiversity and biomass. Additionally, eDNA has been used to detect the locations of the benthic polyp stage [47]; therefore, future studies could be conducted using the eDNA method to investigate the potential polyp habitat in the Bohai Sea during winter when medusae are absent.

### Electronic supplementary material

Below is the link to the electronic supplementary material.

Supplementary Material 1

## Data Availability

Sequence data that support the findings of this study have been deposited in the NCBI database with the primary accession code PRJNA1034232.

## Abbreviations

eDNA: Environmental DNA
BHB: Bohai Bay
LZB: Laizhou Bay
M: The central Bohai Sea area
YR: The Yellow River estuary
N: The Miaodao Archipelago
T: Temperature
S: Salinity
D: Depth
SE: Standard Error
CTD: Conductivity-temperature-depth
GF/F: Glass fiber filters
PO_4_^3−^: Phosphates
SiO_3_^2−^: Silicates
NO_3_^−^: Nitrates
NO_2_^−^: Nitrites
NH_4_^+^: Ammoniums
Chl *a*: Chlorophyll-*a*
PCR: Polymerase chain reaction
FLASH: Fast length adjustment of short reads
QIIME: Quantitative insights into Microbial Ecology
OTUs: Operational taxonomic units
NCBI: National Center of Biotechnology Information
BLAST: Basic Local Alignment Search Tool
ANOVA: Analysis of variation
MEGA: Molecular Evolutionary Genetics Analysis
