# Supplementary material for: Biodiversity and distribution patterns of blooming jellyfish in the Bohai Sea revealed by eDNA metabarcoding
Source: BMC Ecol Evol. 2024 Mar 18;24:37. doi: 10.1186/s12862-024-02224-3 (PMC10946145; doi:10.1186/s12862-024-02224-3)

**Appendix A. Supplementary data.** **Phylogenetic trees of jellyfish based on mitochondrial 16S rRNA gene eDNA profiling from the Bohai Sea.** The scale represents the genetic distance between species.


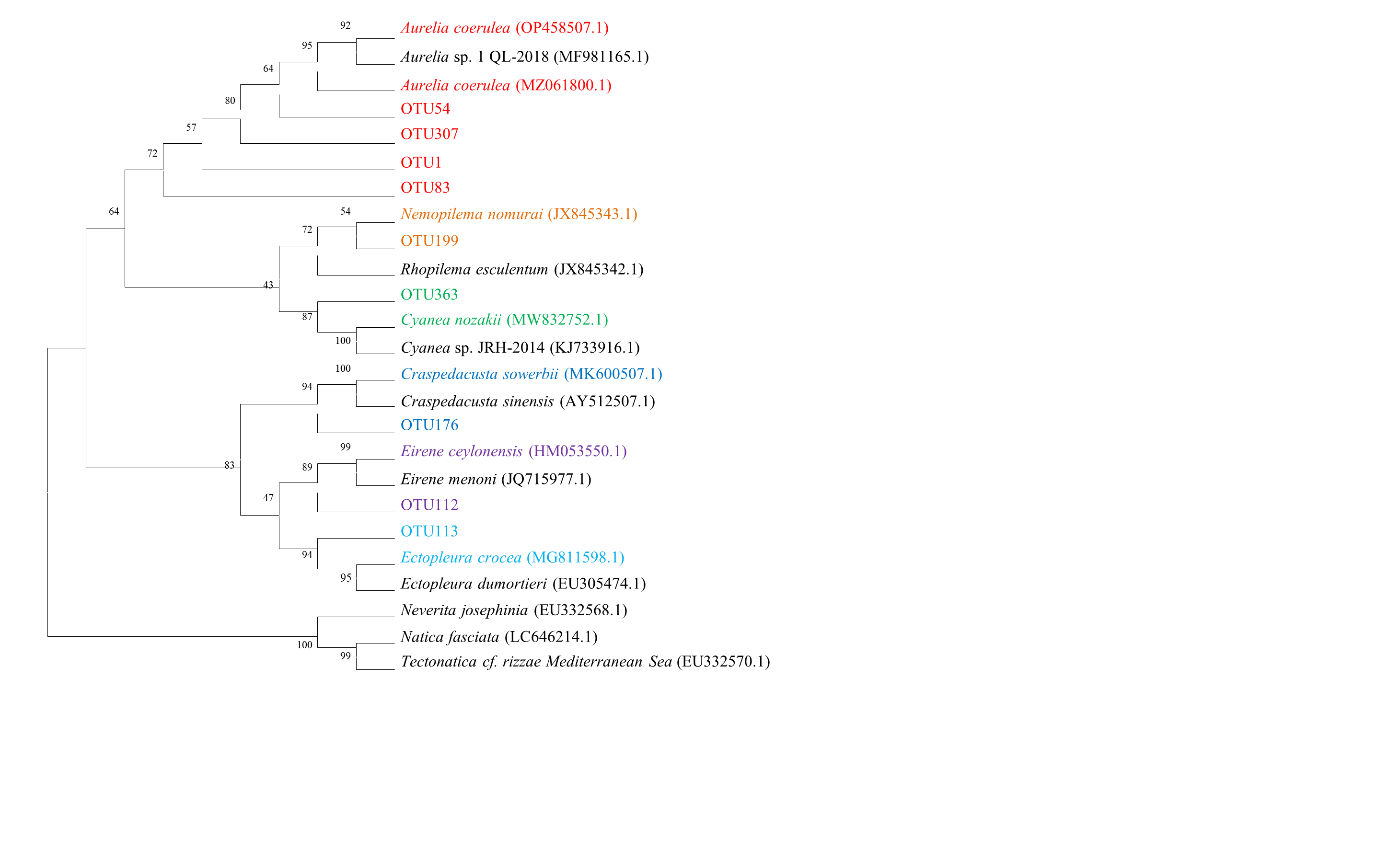

Supplement: Supplementary file 1 — Supplementary Material 1 [file 12862_2024_2224_MOESM1_ESM.docx]
